# New insights into structural and functional relationships between LonA proteases and ClpB chaperones

**DOI:** 10.1002/2211-5463.12691

**Published:** 2019-07-21

**Authors:** Tatyana V. Rotanova, Anna G. Andrianova, Arsen M. Kudzhaev, Mi Li, Istvan Botos, Alexander Wlodawer, Alla Gustchina

**Affiliations:** ^1^ Shemyakin‐Ovchinnikov Institute of Bioorganic Chemistry Russian Academy of Sciences Moscow Russia; ^2^ Protein Structure Section, Macromolecular Crystallography Laboratory National Cancer Institute Frederick MD USA; ^3^ Basic Science Program, Leidos Biomedical Research Frederick National Laboratory for Cancer Research Frederick MD USA; ^4^ Laboratory of Molecular Biology National Institute of Diabetes and Digestive and Kidney Diseases Bethesda MD USA

**Keywords:** AAA^+^ proteins, ATPase module, ClpB chaperones, coiled‐coil fragments, inserted α‐helical domain, Lon protease

## Abstract

LonA proteases and ClpB chaperones are key components of the protein quality control system in bacterial cells. LonA proteases form a unique family of ATPases associated with diverse cellular activities (AAA^+^) proteins due to the presence of an unusual N‐terminal region comprised of two domains: a β‐structured N domain and an α‐helical domain, including the coiled‐coil fragment, which is referred to as HI(CC). The arrangement of helices in the HI(CC) domain is reminiscent of the structure of the H1 domain of the first AAA^+^ module of ClpB chaperones. It has been hypothesized that LonA proteases with a single AAA^+^ module may also contain a part of another AAA^+^ module, the full version of which is present in ClpB. Here, we established and tested the structural basis of this hypothesis using the known crystal structures of various fragments of LonA proteases and ClpB chaperones, as well as the newly determined structure of the *Escherichia coli* LonA fragment (235–584). The similarities and differences in the corresponding domains of LonA proteases and ClpB chaperones were examined in structural terms. The results of our analysis, complemented by the finding of a singular match in the location of the most conserved axial pore‐1 loop between the LonA NB domain and the NB2 domain of ClpB, support our hypothesis that there is a structural and functional relationship between two coiled–coil fragments and implies a similar mechanism of engagement of the pore‐1 loops in the AAA^+^ modules of LonAs and ClpBs.

**Abbreviations**
AAA^+^: ATPases associated with diverse cellular activities
PDB: Protein Data Bank
PQC: protein quality control

The family of Lon proteases, together with several other energy‐dependent proteases, molecular chaperones, as well as regulatory molecules, forms a system of protein quality control (PQC). These proteins play a key role in the maintenance of cellular proteome in all natural kingdoms 1, 2, 3, 4, 5, 6. Chaperones ensure correct folding of the polypeptide products of biosynthesis and participate in the formation of protein assemblies, as well as in preventing aggregation and refolding of modified cellular proteins. ATP‐dependent proteases degrade damaged or abnormal proteins and control the level of regulatory proteins at every stage of the cell cycle.

Most chaperones and all proteases of the PQC system are heat‐shock proteins (Hsp). The PQC proteases are bifunctional oligomeric enzymes. Their proteolytic domains belong to different classes of peptide hydrolases, whereas the ATPase parts are related to the single Hsp100 superfamily denoted AAA^+^ proteins (i.e., ATPases associated with diverse cellular activities) 5, 7, 8, 9, 10, 11, 12. Chaperones‐disaggregases Hsp100 (represented by the ClpB family in bacteria or Hsp104 in eukaryotes) also are part of the same superfamily 7, 8, 9.

The domain organization of AAA^+^ proteins of the PQC system is shown in Fig. 1. The ATPase component of any AAA^+^ protein is a two‐domain AAA^+^ module comprising a large nucleotide‐binding (NB or α/β) domain as well as a small α‐helical (H or α) domain. These modules contain a variety of conserved elements consisting of peptide fragments or, in some instances, single amino acid residues 8, 9, 11, 13. Furthermore, all AAA^+^ proteins include nonhomologous ‘extra’ domains (either located in the protein N‐terminal region or inserted within their NB domains (I domains); see Fig. 1).

**Figure 1:**
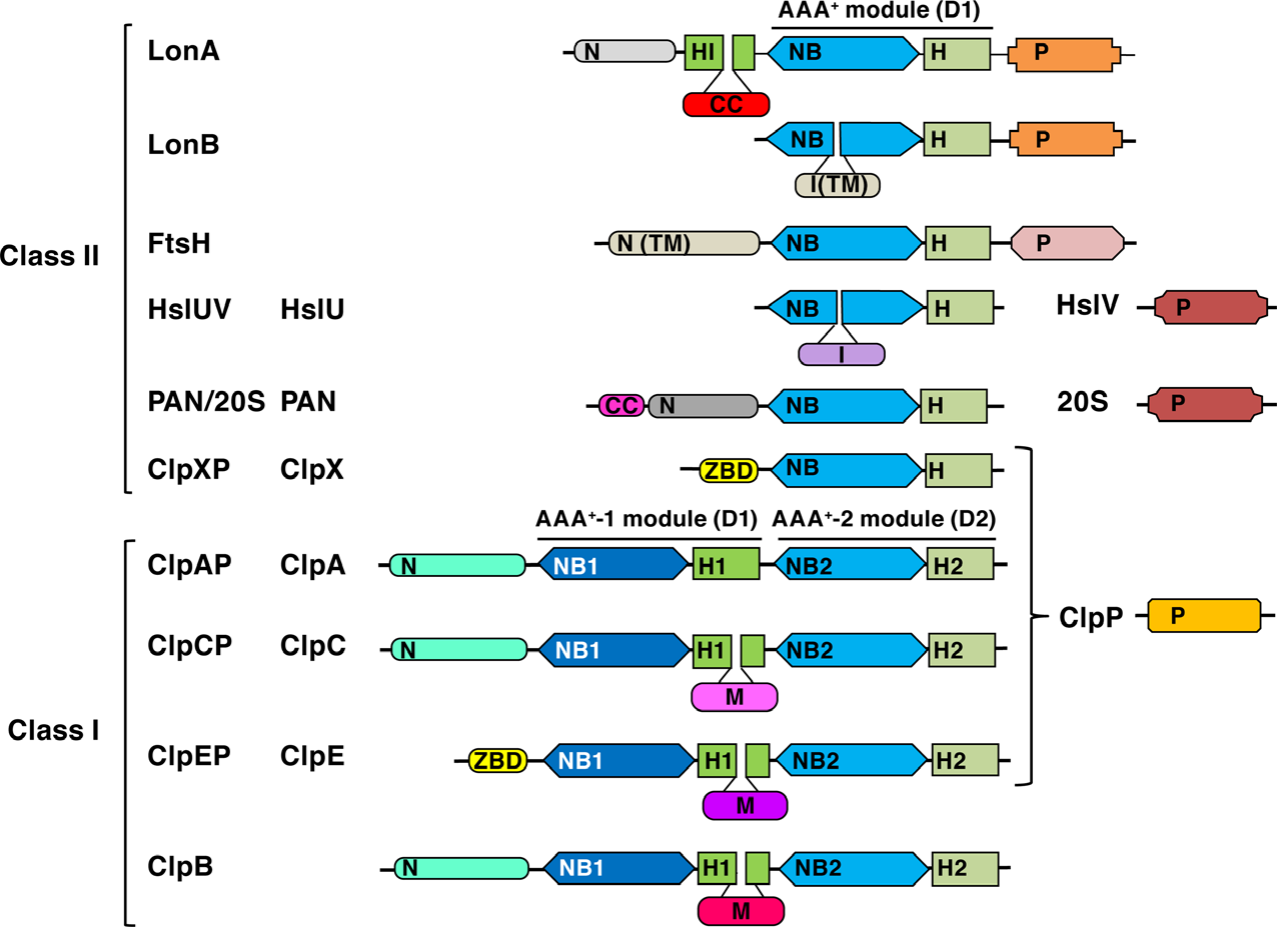
Domain organization in ATP‐dependent proteases and ClpB chaperones of the PQC system. N and ZBD—N‐terminal domains; NB, NB1, and NB2—nucleotide‐binding domains; H, H1, H2, and HI—α‐helical domains; I—inserted domains; TM—transmembrane domains; M—middle CC domains of different sizes, P—proteolytic domains/subunits [clans in MEROPS: SJ (Lon), MA (FtsH), PB (HslV and 20S); SK (ClpP)]; CC‐coiled‐coil regions. Pairs of domains (NB‐H), (NB1‐H1), and (NB2‐H2) form the corresponding AAA^+^ modules.

The AAA^+^ proteins are divided into two classes depending on the number of AAA^+^ modules. The ATPase components of most AAA^+^ proteases and proteolytic complexes (such as proteasomes) that contain only one such module (Lons, FtsH, HslUV, PAN/20S, ClpXP) belong to class II of AAA^+^ proteins, whereas components ClpA, ClpC, and ClpE of other proteases, as well as chaperones‐disaggregases ClpB, have two AAA^+^ modules (D1 and D2) and are assigned to class I (Fig. 1). The H domains of the D1 modules of ClpB, ClpC, and ClpE incorporate inserted coiled‐coil (CC) fragments of different sizes (52 to more than 110 residues). Proteolytic components of the ATP‐dependent proteases can be either domain within a single polypeptide chain of homooligomeric enzymes (Lon and FtsH), or individual subunits of heterooligomeric enzymes (HslUV, ClpXP, ClpAP, and others).

The common pool of Lon proteases comprises a number of different groups, among which the most representative and studied are the subfamilies LonA and LonB 8, 14. Subfamily A includes mainly bacterial and eukaryotic enzymes, whereas most enzymes of the archaeal origin belong to subfamily B. Although proteolytic (P) domains of both, LonA and LonB subfamilies, are serine–lysine hydrolases (clan SJ, family S16 in the MEROPS classification) 15, 16, the environment of the catalytic serine and lysine residues in the P domains of LonA and LonB is different 14. Furthermore, ‘extra’ domains of LonA and LonB proteases are positioned differently. An extra domain in LonA is located at the N terminus of the protein chain, whereas in LonB it is found inside the AAA^+^ module (Fig. 1). It is not yet clear how these differences are reflected in the structures of the full‐length enzymes, as to date no crystal structure of any full‐length Lon protease has been determined. However, crystal structures of a number of fragments consisting of individual domains of LonA and LonB proteases or their combinations are known.

The most prominent feature of LonA proteases, which distinguishes them from proteases of the LonB subfamily, as well as from other AAA^+^ proteins, is a very extended N‐terminal region with 300 to over 400 amino acids. It has been suggested that the N‐terminal region represents a combination of two domains—the actual N‐terminal (N) domain, followed by an inserted domain which is located between the N domain and the AAA^+^ module 17. This inserted domain, referred to as HI(CC) (helical inserted with CC‐fragment 18, Fig. 1), is formed exclusively by α‐helices and is predicted to contain a CC fragment 14, 19 (see also https://embnet.vital-it.ch/software/COILS_form.html). It was suggested that the HI(CC) domain resembles the H1 domain of ClpB chaperone, which also contains a fragment of similar size with CC conformation (namely M domain, Fig. 1) 17, 18.

The role of the N‐terminal region in supporting ATP‐dependent proteolysis and in maintaining the structure of the active enzyme has not yet been fully clarified. There is evidence indicating that this region might play an important role in oligomerization of LonA proteases and could be involved in the binding of protein substrates 20, 21, 22, 23, 24. Furthermore, it was shown that truncations or deletions in the N‐terminal regions of different LonA proteases often disrupt enzymatic activities 20, 21, 22, 25, 26, 27, 28, 29, 30.

With the availability of extensive structural data for the N domain 31, 32, but only incomplete data for the HI(CC) domain 32, 33, 34, we aim here to examine the architecture of the N‐terminal region of LonA proteases, in particular the structure of their HI(CC) domains, focusing on the LonA protease from *Escherichia coli* (*Ec*Lon). The interdomain interactions within monomers and oligomers of *Ec*Lon are also analyzed. As part of this study, we determined the structure of the fragment *Ec*Lon (235–584) that comprises a part of the HI(CC) domain and the AAA^+^ module, since no such structure of *Ec*Lon has been available to date.

## Materials and methods

The amino acid sequences of the chaperones and proteases analyzed in this study were obtained from the UniProt Knowledgebase (http://www.uniprot.org/) and the MEROPS database (www.merops.sanger.ac.uk) 35, respectively. ExPASy server (https://embnet.vital-it.ch/software/ClustalW.html; https://www.ebi.ac.uk/Tools/msa/clustalo/; https://embnet.vital-it.ch/software/COILS_form.html; http://distill.ucd.ie/porter/) was used to evaluate the degree of conservation in sequences, to identify the CC regions in proteins, and for the subsequent topology analysis.

The fragment of *Ec*Lon (235–584) comprising the C‐terminal part of HI(CC) domain and the full‐length AAA^+^ module was produced by limited proteolysis from purified full‐length *Ec*LonA, as previously described 22. As the final step of purification, concentrated protein was loaded on a Superdex 200 Prep grade 26/600 size exclusion column (GE Healthcare, Waukesha, WI, USA) at a flow rate of 0.5 mL·min^−1^. The buffer consisted of 20 mm Tris/HCl pH 7.5, 0.2 m NaCl and 1 mm ADP. For crystallization, the protein was concentrated to 8 mg·mL^−1^ on a Microcon concentrator with 10 kDa MW cutoff (EMD Millipore, Billerica, MA, USA).

Large needle‐shaped crystals were grown by the sitting drop vapor diffusion method with a HydraII crystallization robot (Art Robbins Instruments, Sunnyvale, CA, USA) at 21 °C. The crystals grew in 7–10 days in 30% (w/v) PEG 400, 0.2 m Li_2_SO_4_, 0.1 m Na cacodylate pH 6.5 from the Wizard II screen (Beryllium Discovery, Bedford, MA, USA). Crystals were harvested and flash‐frozen in liquid nitrogen.

X‐ray diffraction data were collected at the SER‐CAT 22‐ID beamline of the Advanced Photon Source at Argonne National Laboratory (Argonne, IL, USA) with a MAR225 detector. The best crystal diffracted to ~ 3.0 Å and belonged to space group P6_1_ or its enantiomorph. Diffraction data were processed with HKL2000 36. Since the completeness and quality of data extending beyond 3.5 Å were poor, we only used data up to that resolution limit (Table 1).

**Table 1:** Data collection and refinement statistics

Data collection	
Wavelength (Å)	1.0
Space group	*P*6_5_
Mol/ASU	1
X‐ray source	APS‐22‐ID
*a*, *b*, *c* (Å)	88.6, 88.6, 67.7
α, β, γ (°)	90, 90, 120
*d*_min_ (Å)^a^	3.5 (3.6–3.5)
Completeness (%)^a^	99.9 (100)
Redundancy^a^	11.1 (11.2)
*R*_merge_ b	0.12 (0.38)
*I*/σ (*I*)^a^	19.2 (6.4)
Refinement	
Resolution	44.3–3.5
Reflections	3856
Atoms	2696
Amino acids	338
*R*_work_ c/*R*_free_ d	0.28/0.32
Validation	
Bond angle RMSD (°)	0.661
Bond length RMSD (Å)	0.003
Ramachandran favored (%)^e^	84.2
Ramachandran allowed (%)^e^	12.5
Clashscore	13.8

a Indicates statistics for the last resolution shell shown in parentheses.

b *R*_merge_ = Σ*_hkl,j_* (|*I_hkl_* − <*I_hkl_*>|)/Σ*_hkl,j_* *I_hkl_*, where <*I_hkl_*> is the average intensity for a set of j symmetry‐related reflections, and *I_hkl_* is the value of the intensity for a single reflection within a set of symmetry‐related reflections.

c *R*_work_ = Σ*_hkl_* (||*F*_o_| − |*F*_c_||)/Σ*_hkl_*|*F*_o_| where *F*_o_ is the observed structure factor amplitude and *F*_c_ is the calculated structure factor amplitude.

d *R*_free_ = Σ*_hkl,T_* (||*F*_o_| − |*F*_c_||)/Σ*_hkl,T_*|*F*_o_|, where a test set, *T*, is omitted from the refinement.

e Calculated with MolProbity 65.

The structure of *Ec*Lon (235–584) was solved by molecular replacement using search models derived from the structures of fragments of LonA proteases from *Meiothermus taiwanensis* [*Mt*Lon; Protein Data Bank (PDB) ID 4YPL 34] and *Bacillus subtilis* (*Bs*Lon; PDB ID 3M6A 33). We obtained the best hit with a homology model built from 4YPL with the Phyre2 server 37. Program phaser 38 from the phenix program suite identified a solution in space group P6_5_. The solution was further improved when the search was performed using separately the NB (α/β) and H (α) domains of the AAA^+^ module. After alternating cycles of refinement with phenix.refine 39 and model building in coot 40, the structure was rebuilt with the help of the crystal structure of *E. coli* H domain (PDB ID 1QZM 41). The structure was refined to an *R*_work_ of 28.4% and *R*_free_ of 32.7% 42 with 86.6% of the residues located in the core region of the Ramachandran plot and 10.7% in the additional allowed region (Table 1). Despite the comparatively low resolution, the electron density is well defined, thus assuring that the overall fold of the structure is correct.

## Results and Discussion

### Crystal structure of the of *Ec*Lon fragment comprising the C‐terminal part of HI(CC) and the AAA^+^ module

**Figure 2:**
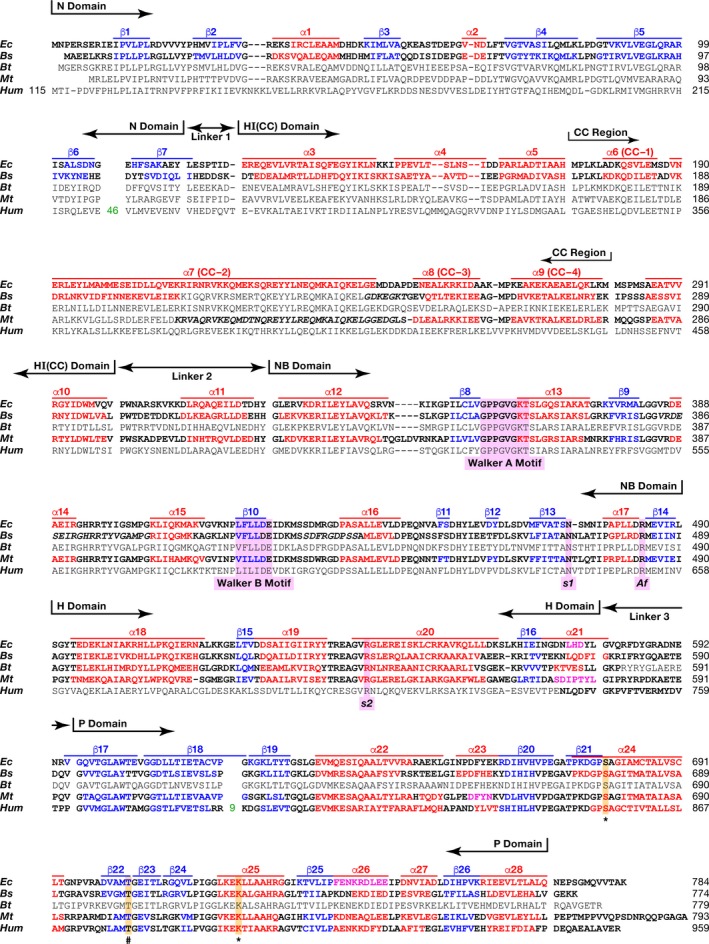
Alignment of the primary and secondary structures of full‐length *Ec*Lon, *Bs*Lon, *Bt*Lon, *Mt*Lon, and *Hum*Lon. Lon proteases are from *Escherichia coli* (*Ec*), *Bacillus subtilis* (*Bs*), *Brevibacillus thermoruber* (*Bt*), *Meiothermus taiwanensis* (*Mt*), and *Homo sapiens* (*Hum*) Domain organization of the enzymes and location of the CC region are shown. Fragments of the primary structures were compared using the program http://www.ch.embnet.org/cgi-bin/clustalw_parser; boundaries were determined based on the secondary structures. Experimentally determined secondary structure elements are shown in bold; fragments of sequences not seen in crystal structures are italicized. Red indicates amino acids that form α helices, magenta—3/10 helices, blue—β strands, and black color indicates amino acids that are not included in the secondary structure elements. Consensus sequence elements are highlighted in lavender: Walker motifs A and B, the residues sensor‐1 (*s1*), sensor‐2 (*s2*), Arg finger (*Af*). The catalytic serine (S*) and lysine (K*) residues as well as important for activity threonine (T^#^) are highlighted in orange.

Members of the LonA protease subfamily comprise five domains within a single chain, connected by three linkers:

$$N-\;\;-\mathrm{linker}\;1-\;\;-\mathrm{HI}\left(\mathrm{CC}\right)-\;\;-\mathrm{linker}\;2-\;\;-\mathrm{NB}-\;\;-H-\;\;-\mathrm{linker}\;3-\;\;-P,$$

where domains N and HI(CC) constitute the N‐terminal region, NB and H form the AAA^+^ module, and P is a serine–lysine peptide hydrolase (Figs 1 and 2A). Structural data are available for only four out of the five domains of LonA proteases from different organisms. They include the functional domains NB, H, P, as well as the N domain from the noncatalytic N‐terminal region.

**Figure 3:**
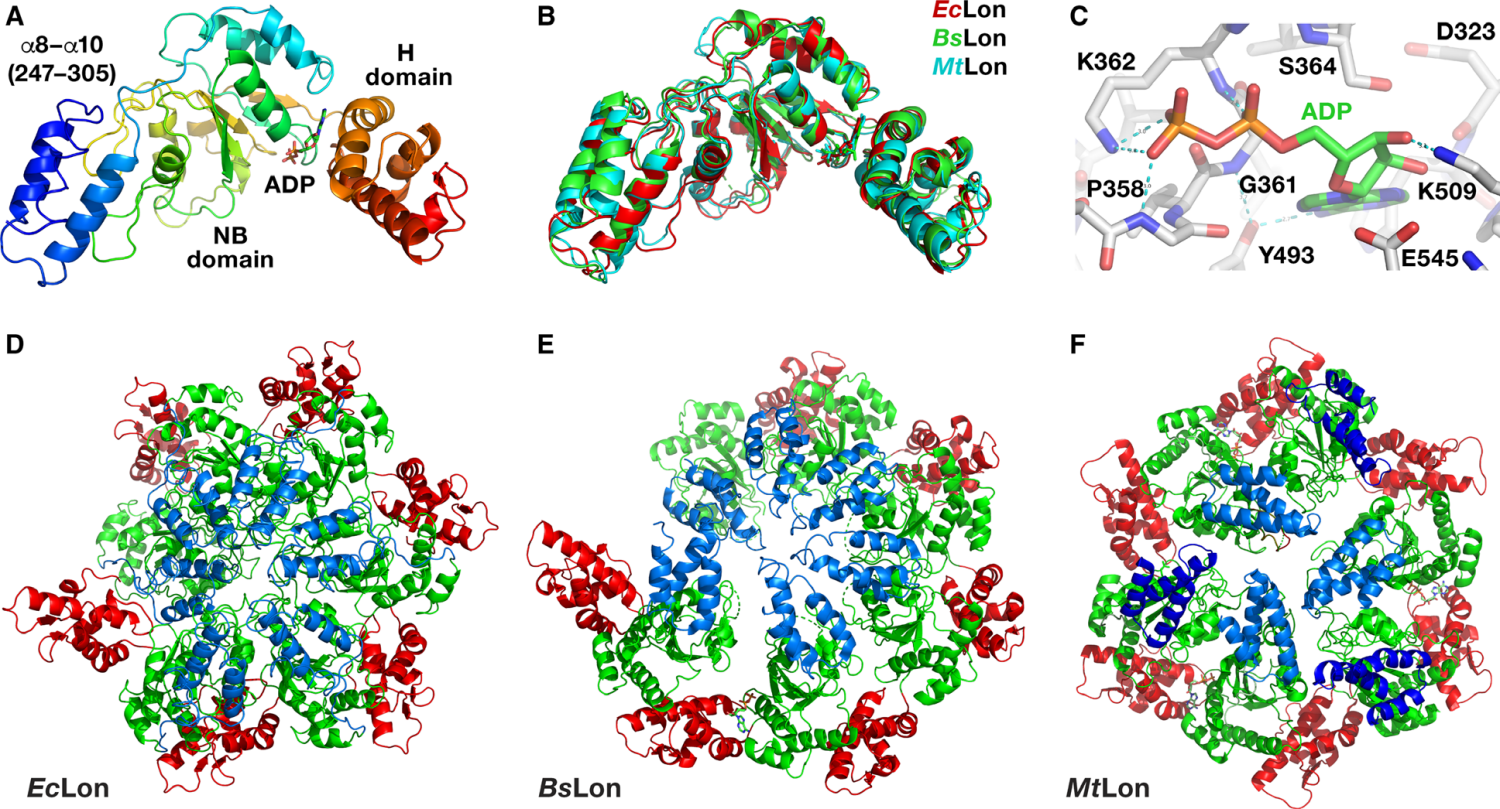
Structures of LonA proteases. (A) Crystal structure of a truncated fragment of *Ec*Lon (residues 235–584 in rainbow coloring). Individual domains are marked. The bound ADP molecule is shown in sticks. (B) Superposition of the crystal structures of three similar fragments of *Ec*Lon (red), *Bs*Lon (green) and *Mt*Lon (cyan). (C) Interactions of ADP molecule in the binding pocket of *Ec*Lon. (D) Cartoon representation of an open ring hexamer in the structure of *Ec*Lon (235–584), with helical arrangement of the monomers around the crystallographic 6_1_ axis passing through the central pore. N‐terminal helices are blue, NB domains are green, and H domains are red. (E) Crystal structure of the open hexamer of *Bs*Lon (240–774; PDB ID 3M6A, P domain not shown for clarity). Colors are as in panel D. (F) Crystal structure of the closed hexamer of the fragment (242–793) of *Mt*Lon (PDB ID 4YPL, P domain not shown for clarity). Two sets of N‐terminal three‐helix bundles located either at the center or at the periphery of a hexamer are shown in light and dark blue, respectively.

Crystal structures of LonA fragments that include the ATPase modules from *Bs*Lon and *Mt*Lon are available 33, 34, but no corresponding structure for *Ec*Lon has been previously reported. We have now determined crystal structure of the fragment of *Ec*Lon comprising the C‐terminal part of HI(CC) and full length of NB and H domains (residues 235–584) at 3.5 Å resolution (PDB ID 6N2I). Although the N‐terminal residue of the construct is Lys235, the first residue visible in the electron density map is Ala247, located before the α8 helix (Figs 2 and 3A). The α12 helix (aa: 331–341) is considered to be the beginning of the NB domain (Fig. 2) 9.

As in all AAA^+^ proteins, the ATPase module of *Ec*Lon is composed of two domains—a large NB (α/β) domain with RecA‐like fold and a small α‐helical (H) domain 8, 9, 11, 43, with an ATP binding site located in the junction between these two domains (Fig. 3A). The overall fold of the AAA^+^ modules of *Ec*Lon, *Bs*Lon, and *Mt*Lon is very similar (Fig. 3B). The structure of the NB domain of *Ec*Lon is characterized by a central β‐sheet comprising five parallel β strands (with strand order 51432), flanked by α helices, while the H domain adapts a four‐helix bundle fold.

A set of conserved consensus fragments of the AAA^+^ module includes Walker motifs A (residues 356–363) and B (419–424), as well as residues forming sensor‐1 (Asn473), sensor‐2 (Arg542), and the arginine finger Arg484 (Fig. 2) 8, 9, 10, 18. All these fragments are crucial for binding and hydrolyzing ATP. The motif A coordinates the γ‐phosphate of ATP, and the motif B coordinates a magnesium ion and activates a water molecule for nucleophilic attack on the γ‐phosphate 43. Sensor‐1 residue coordinates a nucleophilic water molecule, while sensor‐2 interacts with the α‐phosphate of ATP; the arginine finger provides the interactions between neighboring subunits of the enzyme.

An ADP molecule with well‐defined electron density is found in the nucleotide‐binding site (Fig. 3A,C). Lys362 of the consensus Walker A motif (Fig. 2) is involved in interactions with both phosphates of the ADP. Its NZ atom is hydrogen bonded to the β phosphate, whereas its amide interacts with the α phosphate. Another interaction with the β phosphate is provided by the amide nitrogen of Gly359, while orientation of the base is supported by its interactions with Tyr493, and the sugar oxygen interacts with Lys509 (Fig. 3C).

While the fragment of the HI(CC) domain at the N terminus of our structure has poorer density suggesting mobility, the electron density unambiguously defines the overall fold of the NB and H domains of *Ec*Lon, as well as packing of the individual molecules in the oligomer (Fig. 3D). The oligomers in the crystal structures of *Bs*Lon and *Mt*Lon fragments demonstrate two different kinds of hexameric arrangements—hexamers with open and closed rings, respectively (Fig. 3E,F). In our structure of *Ec*Lon, the AAA^+^ modules form an open helical hexameric ring (Fig. 3D), as was observed in the crystal structure of *Bs*Lon (Fig. 3E) 33.

### The unusual N‐terminal region of LonA proteases

Structural data are available for two N‐terminal fragments of *Ec*Lon, consisting of 116 and 245 amino acid residues, respectively 31, 32, as well as for a fragment of *Bs*Lon (1–209) 33. These structures, combined with prediction of the secondary structures for several other LonA proteases (http://www.ch.embnet.org), revealed that the extended N‐terminal region of LonA proteases preceding the AAA^+^ module is formed by two domains, the predominantly β‐structured N domain, and the α‐helical HI(CC) domain (Figs 1 and 2) 17, 18, 32.

In contrast, the ATPase subunits of ClpAP and ClpCP proteases, as well as of ClpB chaperones, have homologous α‐helical N domains 44, 45, 46, whereas subunits ClpX and ClpE (proteases ClpXP and ClpEP) include specific Zn‐binding N domains (ZBD) 47, 48. Membrane‐binding bacterial FtsH proteases contain periplasmic N domains which have α/β‐fold formed by two α‐helices and five β‐strands 49. All these N domains were shown to be involved in recognition and binding of either target proteins or adapter molecules 44, 45, 46, 47, 48. Thus, two‐domain organization of the N‐terminal region radically distinguishes LonA proteases from the other AAA^+^ proteases and ClpB chaperones of the PQC system that bear single N‐terminal domains with varying folds (Fig. 1).

The N domains of bacterial LonAs are composed of seven β strands and two α‐helical fragments (Fig. 2; see also Fig. 1A in 31). A large insert that may include up to 100 amino acids is present in most eukaryotic LonA proteases in the C‐terminal hairpin formed by the sixth and seventh β strands (Fig. 2). The predicted secondary structures of various N domains agree with the experimentally determined secondary structures of the N domains of *Ec*Lon (PDB ID 2ANE ) and *Bs*Lon (PDB ID 3M65), suggesting conservation of the fold. The linker 1 regions between the N domain and the subsequent HI(CC) domain in bacterial and eukaryotic enzymes are of similar size and include 7–8 amino acid residues (Fig. 2).

The second domain in the N‐terminal region of LonA is α‐helical domain, HI(CC), located between the N and NB domains (Fig. 1). HI(CC) domains are similar in size in all LonAs and show a fairly high degree of sequence similarity in groups of bacterial and eukaryotic enzymes (data not shown). A comparative analysis of the primary and the experimental or predicted secondary structures of various LonAs revealed that the HI(CC) domain is formed by eight helices (α3–α10, Fig. 2). According to a prediction (https://embnet.vital-it.ch/software/COILS_form.html), four helices (α6–α9 or CC‐1–CC‐4) represent a CC region, with helix α7 (CC‐2) being unusually long (~ 85 Å, aa V189‐E243 in *Ec*Lon; Fig. 2). It should be emphasized that the degrees of similarity of the CC regions exceed those of the full‐sized HI(CC) domains, and the most conserved fragment of the CC regions is the extended helix α7 (CC‐2).

**Figure 4:**
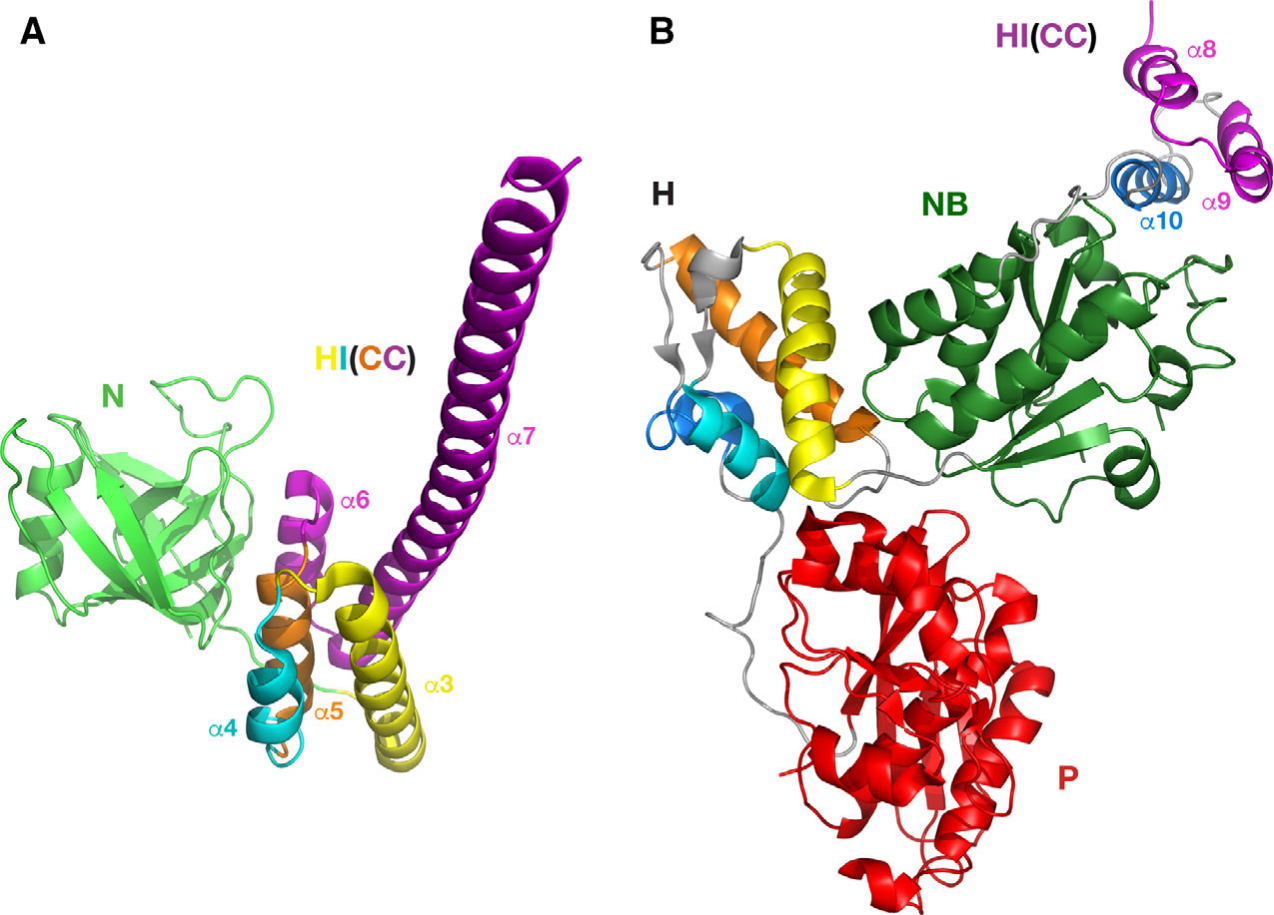
Crystal structures of the fragments *Ec*Lon (1–245) and *Bs*Lon (240–774). (A) N domain of *Ec*Lon (PDB ID 3LJC; residues 1–116) is shown in green; the first five helices of the HI(CC) domain are yellow (α3, residues 124–145), blue (α4, residues 149–159), orange (α5, residues 162–172), and magenta (α6 and α7, residues 181–185 and 189–243), respectively. (B) The last three helices α8–α10 of the HI(CC) domain of *Bs*Lon (PDB ID 3M6A, three N‐terminal helices of the construct) are shown in magenta (α8, α9) and dark blue (α10); the NB domain is green; helices in the H domain are shown with the same color scheme as the first three helices and the last helix of HI(CC) domain, respectively; the P domain is red.

**Figure 5:**
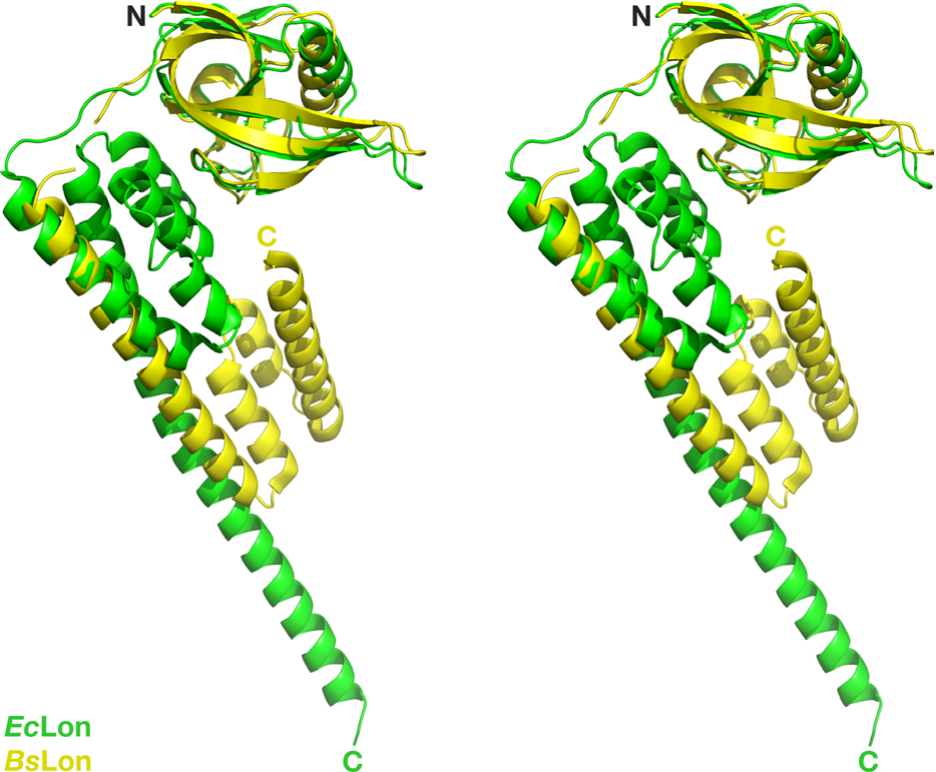
Superposition of the structures of the N‐terminal fragments of *Ec*Lon and *Bs*Lon. The structures of *Ec*Lon (1–245; PDB ID 3LJC, green) and *Bs*Lon (1–209; PDB ID 3M65, yellow) are superimposed by aligning Cα atoms in their N domains.

The packing of the individual helices in the HI(CC) domain is still unknown, as no structure containing an intact HI(CC) domain has been determined. However, the helical nature of the HI(CC) domain is confirmed by combining data from the independently obtained crystal structures of *Ec*Lon (1–245) 32 that includes the N domain and the first five α helices of the HI(CC) domain (Fig. 4A), and the structure of *Bs*Lon (240–774) 33 that comprises the three C‐terminal helices of the HI(CC) domain, AAA^+^ module, and P domain (Fig. 4B). Considering the extensive sequence similarity between *Ec*Lon and *Bs*Lon that exceeds 86%, it might be expected that a hybrid model of a full‐length bacterial LonA could be assembled from these two structures that contain four overlapping residues (Fig. 2). However, in practice such modeling cannot be done due to the large discrepancy in the size and packing of some individual helices comprising the HI(CC) fragment in the structures of differently truncated molecules of *Ec*Lon and *Bs*Lon. A simple structural comparison of the N‐terminal fragments of *Ec*Lon and *Bs*Lon illustrates that phenomenon very clearly (Fig. 5). The sizes of the first helices of HI(CC) domains in these two structures, as well as the topological arrangement of the following helical fragments, are different. Although the variation in the size of the helices and their packing within the HI(CC) fragments could be a result of differences in crystallization or truncation (Fig. 5), it emphasizes the extreme flexibility of this region, substantiating the difficulties encountered in attempts to grow crystals of the full‐length enzyme.

These conclusions are supported by the recently determined structures of two fragments of *Mt*Lon comprising different C‐terminal parts of the HI(CC) domain (PDB IDs 4YPL and 4YPN) 34, as well as by the structure of *E*cLon (235–584) presented here. Although the starting residues in the two fragments of *Mt*Lon are different (residue 207 in 4YPN and residue 242 in 4YPL), the segment comprising residues 207–242 in 4YPN is not visible. Similarly, residues 235–246 are not seen in the structure of *Ec*Lon (235–584). These results confirm the extensive flexibility of this region in the truncated constructs of LonAs.

The HI(CC) domain resembles very closely the H1 domain of first AAA^+^ module of ClpB chaperone with an inserted coiled‐coil M domain, with more than 30% sequence similarity between them. One of the helices in the M domain is composed of 58 residues, similar to the long α7 helix in the *E*cLon HI(CC) domain. This resemblance prompted a conjecture that the HI(CC) domain might correspond to an α‐helical domain of a hypothetical AAA^+^ module that has lost its own NB domain, and is embedded between the N domain and the existing single AAA^+^ module of LonA 18. This conjecture is supported by the prior observation that organization of the sole ATPase modules in the class II AAA^+^ proteins (ClpX and HslU) is similar to the organization of the D2 modules in the class I AAA^+^ proteins 13, 50, 51, 52, 53.

### Structural comparison of the HI(CC) domain of LonA and the H1(M) fragment of ClpB

**Figure 6:**
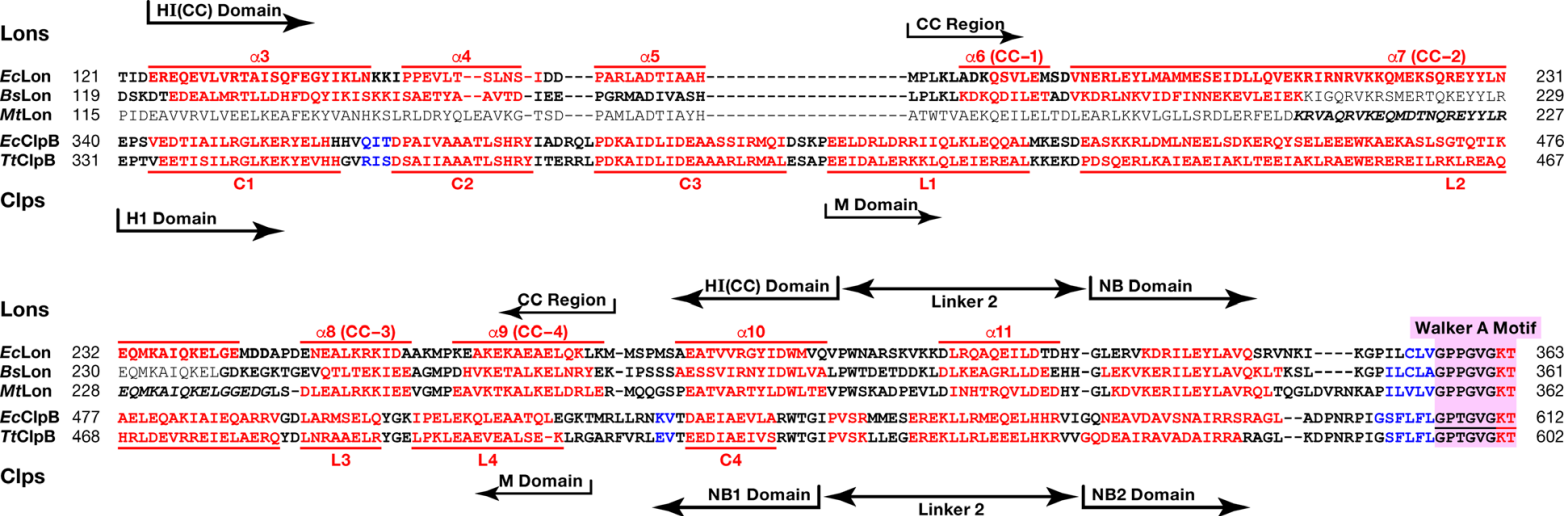
Alignment of the primary and secondary structures of the HI(CC) domains of *Ec*Lon, *Bs*Lon, and *Mt*Lon with the H1(M) fragments of *Ec*ClpB and *Tt*ClpB. The secondary structure elements are designated and highlighted as in Fig. 2. Helices α3–α10 form the Lon HI(CC) domain, helices C1–C4 form the ClpB H1 domain, and helices L1–L4 form the ClpB M domain. Helices in Clp structures are labeled as in original report 46.

Whereas a resemblance of the HI(CC) domain of LonA to the H1(M) fragment of ClpB has been previously suggested 18, no structural basis for such a topological similarity has been established. A comparison of the primary and secondary structures of the HI(CC) domains of *Ec*Lon, *Bs*Lon, and *Mt*Lon on the one hand, and the H1(M) fragments of *E. coli* and *Thermus thermophilus* ClpB chaperones (*Ec*ClpB and *Tt*ClpB) 46 on the other hand (Fig. 6), reveals noticeable similarity. Based on the sequence alignment, it was suggested that eight helices of the HI(CC) domain [five helices (α3–α7) seen in the *Ec*Lon (1–245) and three N‐terminal helices (α8–α10) from the structures of *Ec*Lon (235–584), *Bs*Lon (240–774), and *Mt*Lon (242–793)], might correspond to the C1–C4 helices of the H1 domain of the chaperone, combined with the helices L1–L4 of the inserted M domain (Fig. 6). The fragment (302–324) of *Ec*Lon that includes helix α11 and the equivalent fragments of *Bs*Lon and *Mt*Lon correspond to the linker region connecting the H1 and NB2 domains in *Ec*ClpB and *Tt*ClpB.

**Figure 7:**
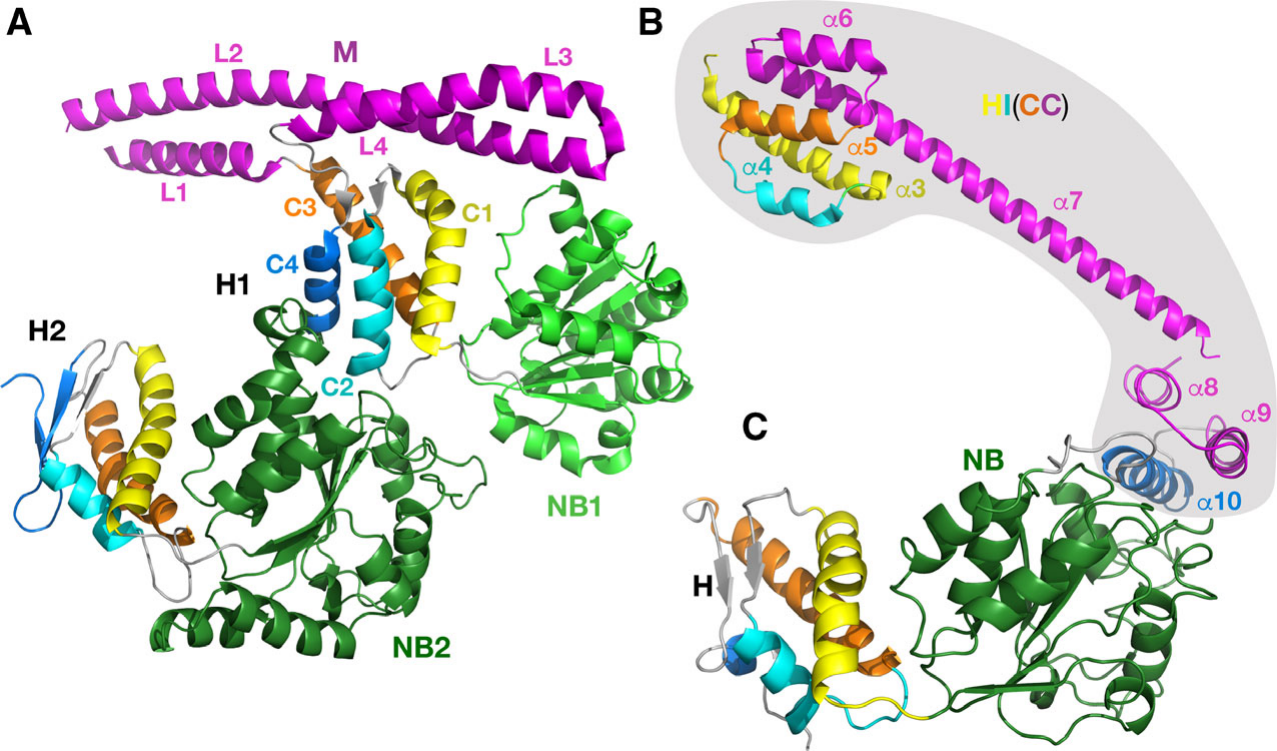
Crystal structures of fragments of *Tt*ClpB chaperone and *Ec*Lon. (A) *Tt*ClpB chaperone (150–854; PDB ID 1QVR); (B) *Ec*Lon (124–245; PDB ID 3LJC); (C) *Ec*Lon (235–584; PDB ID 6N2I). The nucleotide‐binding domains are colored in light (NB1, *Tt*ClpB) and dark green (NB2, *Tt*ClpB and NB, *Ec*Lon). Corresponding helices in the H1 and H2 domains of *Tt*ClpB, as well as in HI(CC) and H domains of *Ec*Lon, are colored identically.

The long α7 (CC‐2) helix, consisting of 55 residues, is a distinctive feature of *Ec*Lon (1–245) fragment (Fig. 4A, magenta). A helix of similar length (L2) is the key element of the propeller‐like M domain of ClpB chaperone (magenta in Fig. 7A) that has CC conformation and is inserted in the chaperone H1 domain (Figs 1 and 6) between its α helices C3 and C4 46. The degree of similarity of these helices in *Ec*Lon and *Ec*ClpB (Fig. 6) is > 45%. An important addition to the similarities listed above is the conservation of consensus elements of AAA^+^ proteins—positively charged ‘sensor‐2’ residues at the beginning of the third helix (Arg164 in *Ec*Lon, Arg162 in *Bs*Lon, and Lys388 in *Ec*ClpB), as well as the Walker A motifs located in the following nucleotide‐binding domains (Fig. 6).

We postulate that the first three helices (α3–α5), combined with the eighth helix (α10) of the HI(CC) domain, form a putative α‐helical domain in the N‐terminal part of the LonA proteases (α‐helical inserted domain, HI domain). Such a domain would topologically resemble the H1 domain of the first AAA^+^ module of ClpB (D1), since it includes the inserted CC region formed by four helices (α6–α9 or CC‐1–CC‐4), equivalent to the arrangement of the helices L1–L4 in the M domain of ClpB. In order to clarify this proposition in structural terms, we marked with identical colors the corresponding helices in the structure of the fragment (150–854) of *Tt*ClpB (PDB ID 1QVR, Fig. 7A) and in the structure of the fragments of *Ec*Lon (124–245) and *Ec*Lon (235–584; Fig. 7B,C). The fragments of *Ec*Lon (124–245) and *Ec*Lon (247–299), highlighted by a gray background in Fig. 7B,C, comprise the structure of the HI(CC) domain.

As mentioned earlier, the mutual arrangements of the helices in HI(CC) in respect to the other domains in the partial structures of *Ec*Lon and *Bs*Lon might be affected by truncations introduced into the constructs that have been used for crystallization. In the absence of a LonA structure with the intact HI(CC) domain, the existing structural fragments containing HI(CC) helices cannot be properly compared between themselves and to ClpB. Therefore, in order to test this hypothesis using the available structural data, we analyzed the resemblance between the two proteins on the level of both the secondary and tertiary structures, comparing either the individual helices, or the whole domains.

**Figure 8:**
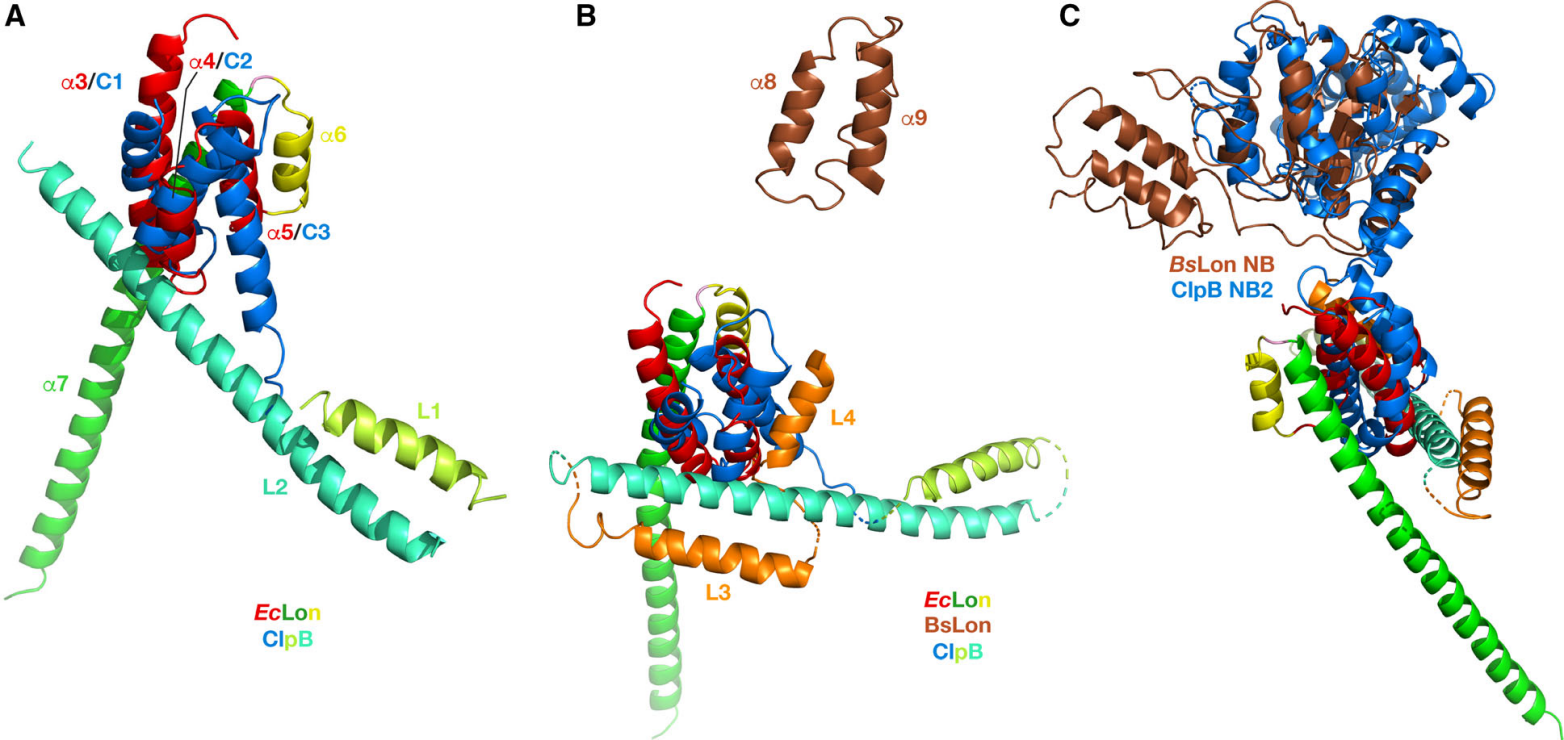
Structural comparison of the HI(CC) domain of LonAs and H1 domain of *Ec*ClpB. (A) The first three helices of the *Ec*Lon HI(CC) domain (α3–α5, red, PDB ID 3LJC) are superimposed on the first three helices of the H1 domain of *Ec*ClpB (blue, PDB ID 4D2U). Two following helices in both proteins are colored in different shades of yellow and green, correspondingly. (B) Topological assignment of the remaining three helices of the HI(CC) domain. Helices α8–α10 of the *Ec*Lon HI(CC) domain (brown, PDB ID 6N2I) are placed within the frame of Fig. 8A by superimposing the NB domain of *Ec*Lon and NB2 domain of *Ec*ClpB. Corresponding helices in ClpB are shown in orange. (C) Flexible linker region between the N‐terminal three‐helix bundle and NB domain in *Ec*Lon (brown). The NB2 domain of *Ec*ClpB, superimposed with the latter, is shown in blue.

We started by superimposing selected helices comprising the HI(CC) domain of *Ec*Lon with their counterparts in the H1 domain of the first AAA^+^ module of ClpB 54 (Fig. 8). As can be seen in Fig. 8A, the first three helices of the LonA HI(CC) domain (α3–α5) could be superimposed quite well onto the corresponding helices of the H1 domain of ClpB (C1–C3). The short helix α6, preceding the long helix α7 in the structure of the N‐terminal fragment of *Ec*Lon (yellow in Fig. 8A), is oriented differently than the corresponding first helix L1 of ClpB M domain (shown in a different shade of yellow), which leads to a dramatic difference in the orientation of the long helices α7 and L2 (shown in two different shades of green).

Furthermore, helices α8 (CC‐3), α9 (CC‐4), and α10 at the N terminus of *Ec*Lon (235–584) correspond to helices L3, L4, and C4 of ClpB (Figs 6 and 8B, shown in brown and orange, respectively). In the hexamers of *Ec*Lon (235–584) and *Bs*Lon (240–774), these helices are oriented toward the central pore (shown in blue in Fig. 3D,E). The authors attribute the open ring arrangement of the monomers in the hexamer of *Bs*Lon to the observed position of this helical bundle in the crystal structure that ‘is incompatible’ with formation of a closed ring 33. The helices α8–α10 in *Ec*Lon are connected to the NB domain via a long, extended linker (Fig. 8C, colors as above), which would permit flexibility in the relative positioning of these helical fragments in truncated molecules of *Ec*Lon and *Bs*Lon. That conclusion is also supported by variations in the relative position of the N‐terminal three‐helix bundle in six monomers, as well as by increased temperature factors for this area. Therefore, the observed localization of the N‐terminal helices of *Ec*Lon and *Bs*Lon may not reflect their position in the structure of a Lon molecule in which the HI(CC) domain would be intact.

Two crystal structures of the closed ring hexamers of the fragments (242–793) and (207–492) of *Mt*Lon (PDB ID 4YPL, 4YPN) with a similar three‐helix bundle at the N terminus of the molecule also indicate that formation of a closed hexameric ring does not tolerate the presence of all three‐helix bundles in the central pore, but requires to move three bundles out of six to the periphery of the ring (Fig. 3F).

### Similarities and differences in the structures of the NB domains in AAA^+^ modules of LonA proteases and Clp chaperones

Structural data on the NB domains of LonA proteases include our structure of *Ec*Lon presented here (Fig. 3A), as well as two high‐resolution crystal structures of *Bs*Lon (PDB ID 3M6A, Fig. 4B) 33 and *Mt*Lon (PDB ID 4YPN) 34. The overall fold of the LonA NB domain is typical of many other AAA^+^ proteins, in particular of the ATPase components of ClpXP (PDB ID 3HTE) 55 and ClpAP proteases (PDB ID 1KSF) 44, as well as both NB domains of ClpB chaperones (PDB IDs 1QVR, 1JBK) 46, 56.

Similarly to other AAA^+^ ATPases, LonA proteases are expected to function as hexamers with a central pore that is lined with axial loops, required for protein unfolding and translocation. Three different pore loops, called ‘GYVG’ (or pore‐1), pore‐2 and ‘RKH’, are usually found in almost all AAA^+^ unfoldases 55, 57.

**Figure 9:**
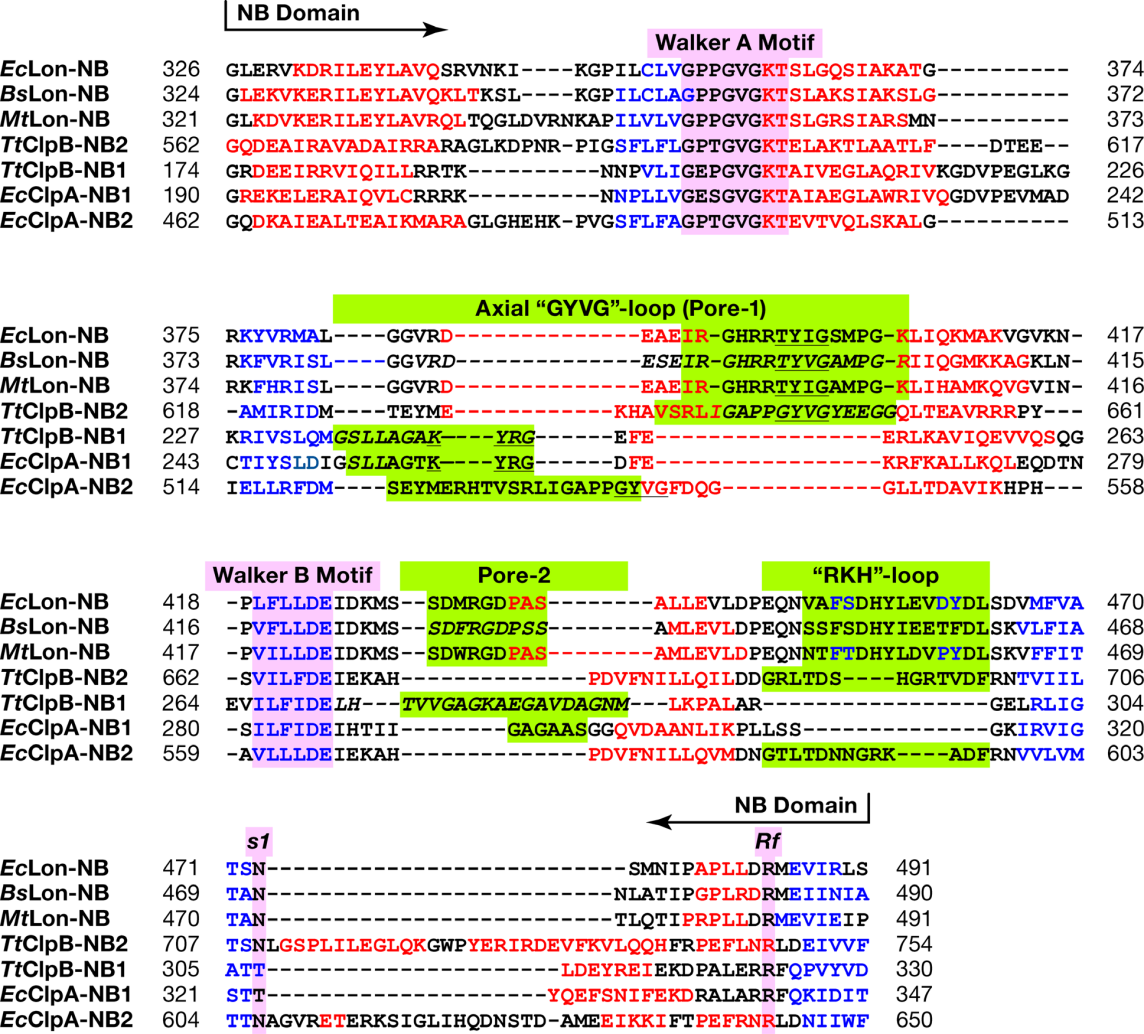
Structure‐based sequence alignment of the NB domains of *Ec*Lon, *Bs*Lon, and *Mt*Lon with the NB1 and NB2 domains of *Ec*ClpA and *Tt*ClpB chaperones. The secondary structure elements are designated and highlighted as in Fig. 2. Axial pore loops are highlighted in green.

**Figure 10:**
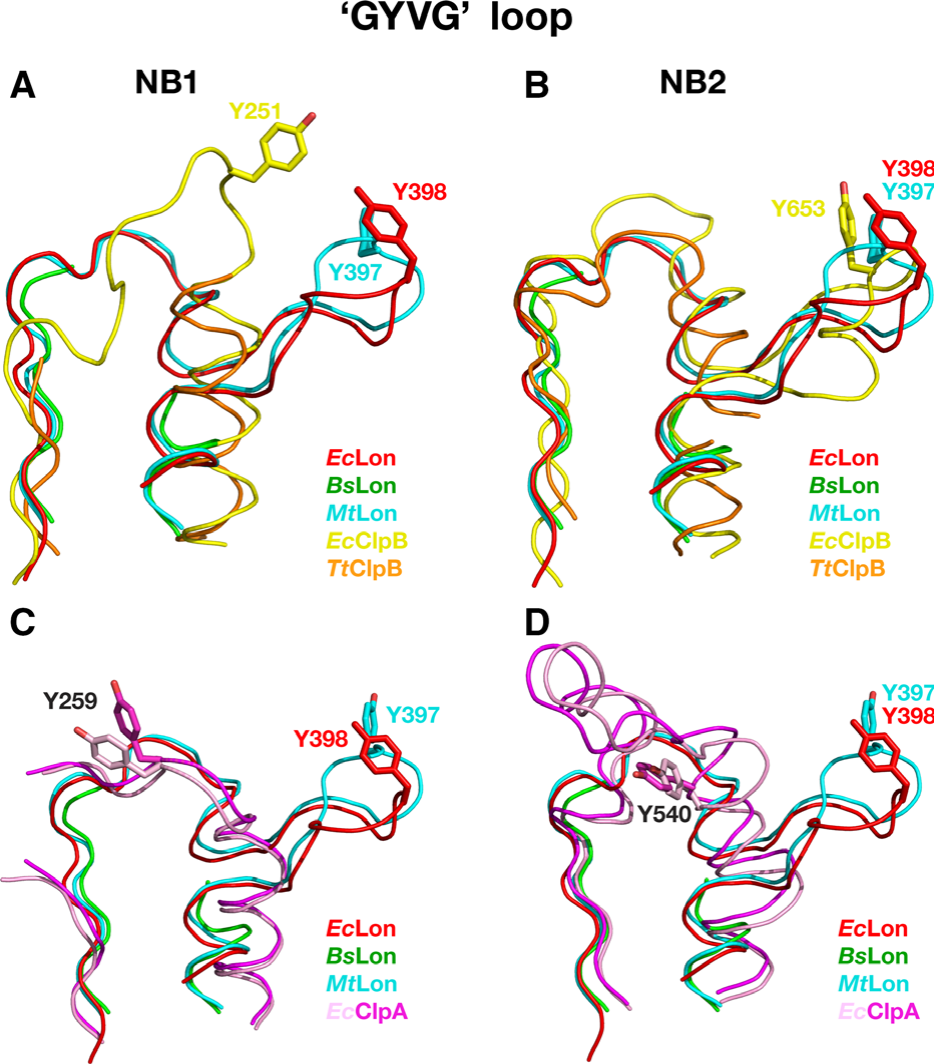
A comparison of the fragments containing axial GYVG (pore‐1) loops in the crystal structures of NB domains of LonAs and NB1–2 domains of ClpA and ClpB chaperones. GYVG comprising fragments from *Ec*Lon (PDB ID 6N2I, residues 378–409, Tyr398 shown in sticks, red), *Bs*Lon (PDB ID 3M6A, residues 376–408 with a break between residues 382 and 403, green) and *Mt*Lon (PDB ID 4YPL, residues 377–409, Tyr397 shown in sticks, blue) are superimposed: (A) with corresponding fragments from the NB1 domains of *Ec*ClpB (PDB ID 5OFO, residues 237–267, Tyr251 shown in sticks, yellow) and *Tt*ClpB (PDB ID 1QVR, residues 230–259 with a break between 234 and 246, orange); (B) with corresponding fragments from the NB2 domains of *Ec*ClpB (PDB ID 5OFO, residues 628–668, Tyr653 shown in sticks, yellow) and *Tt*ClpB (PDB ID 1QVR, residues 620–656 with a break between 636 and 651, orange); (C) with corresponding fragments from the NB1 domains of *Ec*ClpA (PDB ID 1KSF, residues 246–273 with a break between 251 and 255, Tyr259 shown in sticks, pink) and *Ec*ClpA (PDB ID 1R6B, residues 246–273 with a break between 251 and 255, Tyr259 shown in sticks, magenta); (D) with corresponding fragments from the NB2 domains of *Ec*ClpA (PDB ID 1KSF, residues 517–552, Tyr540 shown in sticks, pink) and *Ec*ClpA (PDB ID 1R6B, residues 517–552, Tyr540 shown in sticks, magenta).

The homologs of GYVG (pore‐1) loops are found in all AAA^+^ proteases and chaperones of the ClpB/Hsp104 family 55, 57, 58, 59, 60. As currently established 57, 61, GYVG loops are involved during all stages of molecular machinery work. They interact with polypeptide substrates and pull them into the pore in a concerted way with nucleotide‐dependent loop movements unfolding those substrates that cannot enter the pore otherwise. A comparison of the fragments containing GYVG loops in the structures of AAA^+^ modules of chaperones ClpA and ClpB revealed a distinct difference in the location of the functional motif within the individual loops in their NB1 and NB2 domains. Structure‐based sequence alignment of the NB domains of LonA proteases with the NB1 and NB2 domains of ClpA and ClpB chaperones (Fig. 9) indicates variability in the placement of GYVG motif in these proteins. A structural superposition of the corresponding fragments, comprising GYVG signature motives in these proteins (Fig. 10), provides a good illustration of this feature.

A pairwise comparison of the fragments containing the GYVG loops in the structures of NB domains of *Ec*Lon, *Bs*Lon, and *Mt*Lon with those in the NB1 and NB2 domains of ClpA and ClpB chaperones reveals a striking similarity in the location of GYVG motif with a conserved tyrosine residue within the helical fragment of these loops between the LonA NB and ClpB NB2 domains (Figs 9 and 10B). It must be stressed that although the pore‐1 loop is partially disordered in the NB1 domain of *Tt*ClpB, GYVG location is unambiguously different in both, *Ec*ClpB and *Tt*ClpB, from the one described above (Figs 9 and 10A). It is reminiscent of the position of the GYVG loop in *Ec*ClpA NB2 domain, although the placement of the conserved tyrosine is different among them (Figs 9 and 10D). It should be stressed, however, that the conformation of the GYVG loop in the *Ec*ClpA NB2 domain might be affected by the presence of a Mg ion bound in the vicinity of this loop in the crystals 62. On the other hand, location of the conserved tyrosine residues in the NB1 domains of ClpA and ClpB is similar (Figs 9 and 10A,C).

Our analysis indicates that there is a correlation between the presence of the CC fragment, embedded in the α‐helical domain that precedes either the single or second AAA^+^ module, and the location of GYVG motif in the pore‐1 loop of the following NB domain. Other proteins with a single AAA^+^ module (ClpX, HslU, FtsH) that do not contain a CC insertion have their GYVG signature located on the top of the loop, as shown in Fig. 10A.

**Figure 11:**
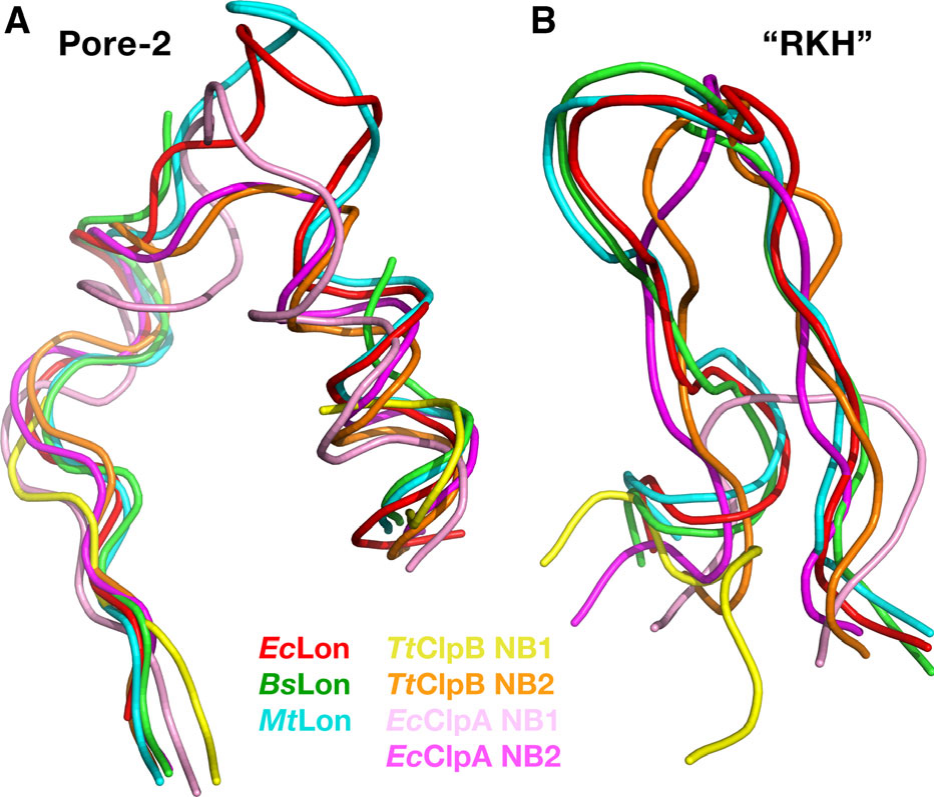
A comparison of the fragments containing axial loops pore‐2 and RKH in the crystal structures of the NB domains of LonAs and NB1–2 domains of ClpA and ClpB chaperones. (A) Pore‐2 comprising fragments from *Ec*Lon (PDB ID 6N2I, residues 419–445, red), *Bs*Lon (PDB ID 3M6A, residues 416–442 with a break between 427 and 437, green), and *Mt*Lon (PDB ID 4YPL, residues 417–443, blue) are superimposed onto corresponding fragments from *Tt*ClpB (PDB ID 1QVR) of both the NB1 domain (residues 264–295 with a break between 271 and 291, yellow), and the NB2 domain (residues 662–684, orange), as well as with the corresponding fragments from *Ec*ClpA (PDB ID 1R6B) of both the NB1 (residues 280–307, pink), and NB2 domains (residues 559–581, magenta); (B) RKH comprising fragments from *Ec*Lon (PDB ID 6N2I, residues 447–465, red), *Bs*Lon (residues 445–463, green) and *Mt*Lon (residues 446–464, blue) are superimposed onto the corresponding fragments from *Tt*ClpB of both the NB1 (residues 296–302, yellow) and NB2 domains (residues 686–700, orange), as well as with the corresponding fragments from *Ec*ClpA of both the NB1 (residues 308–316, pink), and NB2 domains (residues 582–597, magenta).

Location of two other axial loops (pore‐2 and RKH) is highly conserved in the structures of LonA proteases and of both chaperones, ClpA and ClpB (Fig. 11A,B). Pore‐2 loops are also pointing into the central pore and are expected to interact with the substrate, as pore‐1 loops do, but at a different stage of substrate translocation 55, 59, 63. In LonA proteases, they are more similar in length to their counterparts in the NB1 domains of both ClpA and ClpB (Figs 9 and 11A).

RKH loops are located at the upper entry to the central pore and are expected to interact with the substrate at early stages of its approach to the AAA^+^ molecular machine, stabilizing the contacts during initial complex formation 55, 64. RKH loops of LonA proteases are much more similar in length to their structural equivalents in the NB2 domains of ClpA and ClpB chaperones, than to the shorter ones in their NB1 domains (Figs 9 and 11B).

## Conclusions

The principal aim of this study was to provide structural support for the hypothesis that LonA proteases, which bear a single classical AAA^+^ module, may also contain a part of the second AAA^+^ module, which is present in full in the Clp enzymes of class I AAA^+^ proteins. A comparative analysis of the corresponding domains in the available structures of LonA proteases and ClpB chaperones revealed similarities on all four levels of structural organization of these proteins (from primary to quaternary). There is a strong indication that the architecture of the unique HI(CC) domain of LonA proteases resembles the structure of the H domain of the D1 AAA^+^ module of ClpB chaperones. The number of helices, their topology, and the presence of the long helix in the HI(CC) domain, the structural equivalent of which in the M domain of D1 is engaged in regulation of the necessary dynamic rearrangement of the subunits in a hexamer of ClpB 54, are similar in both proteins. However, discrepancies in the sizes of the corresponding helices would imply that their packing within the individual domains might be different.

The other argument in favor of this hypothesis comes from studies evaluating functional characteristics of an *E. coli* LonA mutant with deleted HI(CC) domain (residues 124–304) 29. The ATPase activity of this mutant was shown to decrease by more than an order of magnitude, and its proteolytic activity was almost totally lost. At the same time, the enzyme retained partial ability to hydrolyze peptide substrates (about 30%), although, unlike intact *Ec*Lon, binding of nucleotides and their complexes with magnesium ions did not affect the efficiency of peptide hydrolysis. It was concluded that the inserted HI(CC) domain is needed for formation of a functionally active enzyme, and the absence of this domain alters coupling between ATP hydrolysis and substrate proteolysis, as well as it affects interactions between LonA and a protein substrate.

This idea is also supported by comparison of the functionally important axial loops in the NB domain of the single AAA^+^ module of LonA with the corresponding ones in the NB1 and NB2 domains of two AAA^+^ modules of ClpA and ClpB chaperones, respectively. Significant results of that analysis are provided for the most conserved axial pore‐1 loops (GYVG) that are engaged in the interactions with substrate during all stages of protein function. A comparison of the fragment comprising this loop in LonA with the corresponding fragments of both the NB1 and NB2 domains of ClpA and ClpB chaperones unambiguously reveals a singular match in the location of the GYVG loop between the LonA NB domain and the NB2 domain of ClpB. This finding suggests that the mechanism of engagement of pore‐1 loops in these AAA^+^ modules of LonA and ClpB might be indeed similar. Our observations, combined with the data derived from the structure of ClpB with bound substrate 61 that designate the AAA^+^‐2 module as being the main motor of ClpB, give a new perspective to a functional meaning of the resemblance between the sole ATPase module of class II and the AAA^+^‐2 module of class I of the AAA^+^ proteins, and also correlate with the matching topology between the HI(CC) domain of LonAs and H1 domain of ClpBs.

In the absence of a structure of full‐length LonA protease, the structural elements that carry the functional or regulatory properties for these molecular machines cannot be accurately matched up between different partial structures. However, available structural data strongly support the hypothesis of their revised structural and functional relationship, thus suggesting that LonA proteases, exhibiting the structural features of both classes of AAA^+^ proteins, may represent a novel subclass of AAA^+^ proteins, distinctive from the established class I and class II.

## Conflict of interest

The authors declare no conflict of interest.

## Author contributions

TVR and AG conceived this study and, together with AW, supervised its conduct. IB and ML performed the crystallographic experiments. AGA and AMK performed sequence alignments and generated the illustrations. All authors analyzed and discussed the data and contributed to writing the manuscript.
